# Treatment-emergent central sleep apnea in a patient with multiple system atrophy: case report

**DOI:** 10.1016/j.bjorl.2024.101465

**Published:** 2024-07-09

**Authors:** Carolina Almeida Grinfeld, Yasmin de Rezende Beiriz, Thatiana Pereira Silva, Natalia Martins de Araújo, Mirella Pereira Souza Paixão, Julyana Carneiro Gomes, Paulo Sergio Lins Perazzo

**Affiliations:** Hospital Otorrinos de Feira de Santana, Feira de Santana, BA, Brazil

## Introduction

Complex Sleep Apnea or Treatment-Emergent Central Sleep Apnea (ASC-TE) is a rare form of central apnea characterized by the persistence or emergence of central sleep apnea during positive airway pressure therapy for obstructive sleep apnea, which cannot be explained by other causes.^1^ Its prevalence ranges from 0.56% to 20.3% and the triggering factors are still poorly understood.^2^

In this report, we present a case of complex apnea in a patient subsequently diagnosed with multiple system atrophy.

The study was approved by the research ethics committee under registration number 5.667.835.

## Case presentation

Male patient, 57 years old, BMI 23.05 kg/m^2^, Epworth Sleepiness Scale 13, presented with intense nocturnal snoring associated with daytime sleepiness, with excessive impact on work activities. No comorbidities and use of medication. He usually slept at 10 pm and got up at 6 am. When waking up in the morning, I usually had a feeling of dry mouth and unrefreshing sleep. Significant physical examination findings included inferior turbinate hypertrophy, Brodsky grade III tonsil, retrognathia, Mallampati II classification. The main diagnostic hypothesis was Obstructive Sleep Apnea (OSA). Type I polysomnography was performed, with an Apnea-Hypopnea Index (AHI) of 49.3 h, with 49 apnea/hour and 0.3 hypopnea/hour, a markedly increased value, and a NADIR of 66%. The examination also revealed moderate and intermittent snoring, increased latency to REM sleep, decreased percentage of REM sleep, reduced sleep efficiency, and increased brief awakening index.

Once the diagnosis of OSA was confirmed, an examination for CPAP titration was performed. The patient was instructed about sleep hygiene and to start treatment with a pressure of 11 cm H_2_O.

Treatment with nasal CPAP was started and, after one year of treatment, the patient returned reporting nocturnal hypertension and worsening sleepiness despite the adequate use of CPAP. A new titration for CPAP was requested in the sleep laboratory, however, when increasing the pressure in the CPAP to stop the obstructive apnea, apnea of central origin occurred, maintaining a markedly high AHI, configuring the diagnosis of complex apnea. It is worth noting that when the patient began follow-up, he did not present any neurological symptoms. After a year of using CPAP, he still had no symptoms of central disease, but only nocturnal hypertension and drowsiness. The complex apnea could not be attributed to another cause. In complex apnea, central apnea arises in the context of positive airway pressure treatment for obstructive sleep apnea and is not attributable to another cause.

Titration to BiPAP was performed and with IPAP of 11 cm H_2_O and EPAP of 7 cm H_2_O, the patient presented good tolerance and reduction of obstructive and central events. After five years using the BiPAP, he returned complaining of imbalance-type vertigo, ataxia, sialorrhea and dizziness. He was being followed up by a neurologist, with a diagnosis of multiple system atrophy. Magnetic Resonance Imaging (MRI) of the skull revealed bilateral cerebellar and pons atrophy.

## Discussion

The diagnosis of ASC-TE is made through polysomnography. Studies suggest waiting 4‒8 weeks with the use of CPAP before changing treatment, even with central events, as these can be transient.^3^

The diagnosis of complex apnea was made because when the patient presented central apnea when increasing CPAP pressure, he did not present any neurological symptoms or suggestive of Multiple System Atrophy. Neurological symptoms developed five years after the diagnosis of complex apnea.

Therefore, it cannot be considered the evolution of obstructive apnea, but rather complex apnea.

When CPAP fails to correct ASC-TE, in symptomatic patients and when the Apnea Hypopnea Index is >15 at follow-up, effective treatment options include a change in ventilation therapy (adaptive servoventilation or BiPAP) or additional nocturnal oxygen supplementation.^4^

MSA is a progressive neurodegenerative disorder characterized by autonomic failure, parkinsonian features poorly responsive to levodopa, and cerebellar ataxia.^5^ About 15% to 69% of patients with MSA may present with respiratory disorders, such as obstructive sleep apnea, and central sleep apnea.^5^ These symptoms are commonly found in later stages of the disease, but may be the main feature in some cases.

The natural course of the disease affects the brainstem and its centers for regulating breathing, which can lead patients to central apnea. In this case, CPAP therapy may not be beneficial and use of BiPAP and servo-adaptive ventilation is recommended.^5^

## Conclusion

The patient was a unique case due to complaints of sleep disturbance before actual neurological manifestations. In addition, the rarity is still due to the presentation of central apnea, and not obstructive apnea, as in most cases in MSA. At diagnosis, the patient did not present any neurological symptoms, which developed only five years later, which justifies the term central apnea, as the event was not attributable to another cause.

He was followed up for years, initially adapting well to the treatment of OSA with CPAP. However, at each new consultation, she presented worsening in some aspect, not only the apnea, but also neurological alterations, which led to the suspicion of an underlying disease of a neurodegenerative nature. This case demonstrates the importance of the otorhinolaryngologist in identifying sleep disorders and assessing the patient globally.

## Approval

All authors approved the manuscript. All authors read and approved the final version submitted to Brazilian Journal of otorhinolaryngology.

## Conflicts of interest

The authors declare no conflicts of interest.
